# Are *Treponema pallidum* Specific Rapid and Point-of-Care Tests for Syphilis Accurate Enough for Screening in Resource Limited Settings? Evidence from a Meta-Analysis

**DOI:** 10.1371/journal.pone.0054695

**Published:** 2013-02-26

**Authors:** Yalda Jafari, Rosanna W. Peeling, Sushmita Shivkumar, Christiane Claessens, Lawrence Joseph, Nitika Pant Pai

**Affiliations:** 1 Department of Epidemiology, Biostatistics and Occupational Health, McGill University, Montréal, Canada; 2 London School of Hygiene and Tropical Medicine, London, United Kingdom; 3 Institut national de santé publique (INSPQ), Montréal, Canada; 4 Division of Clinical Epidemiology, Department of Medicine, McGill University and MUHC, Montréal, Canada; University of Ottawa, Canada

## Abstract

**Background:**

Rapid and point-of-care (POC) tests for syphilis are an invaluable screening tool, yet inadequate evaluation of their diagnostic accuracy against best reference standards limits their widespread global uptake. To fill this gap, a systematic review and meta-analysis was conducted to evaluate the sensitivity and specificity of rapid and POC tests in blood and serum samples against *Treponema pallidum* (TP) specific reference standards.

**Methods:**

Five electronic databases (1980–2012) were searched, data was extracted from 33 articles, and Bayesian hierarchical models were fit.

**Results:**

In serum samples, against a TP specific reference standard point estimates with 95% credible intervals (CrI) for the sensitivities of popular tests were: i) Determine, 90.04% (80.45, 95.21), ii) SD Bioline, 87.06% (75.67, 94.50), iii) VisiTect, 85.13% (72.83, 92.57), and iv) Syphicheck, 74.48% (56.85, 88.44), while specificities were: i) Syphicheck, 99.14% (96.37, 100), ii) Visitect, 96.45% (91.92, 99.29), iii) SD Bioline, 95.85% (89.89, 99.53), and iv) Determine, 94.15% (89.26, 97.66). In whole blood samples, sensitivities were: i) Determine, 86.32% (77.26, 91.70), ii) SD Bioline, 84.50% (78.81, 92.61), iii) Syphicheck, 74.47% (63.94, 82.13), and iv) VisiTect, 74.26% (53.62, 83.68), while specificities were: i) Syphicheck, 99.58% (98.91, 99.96), ii) VisiTect, 99.43% (98.22, 99.98), iii) SD Bioline, 97.95%(92.54, 99.33), and iv) Determine, 95.85% (92.42, 97.74).

**Conclusions:**

Rapid and POC treponemal tests reported sensitivity and specificity estimates comparable to laboratory-based treponemal tests. In resource limited settings, where access to screening is limited and where risk of patients lost to follow up is high, the introduction of these tests has already been shown to improve access to screening and treatment to prevent stillbirths and neonatal mortality due to congenital syphilis. Based on the evidence, it is concluded that rapid and POC tests are useful in resource limited settings with poor access to laboratories or screening for syphilis.

## Introduction

The World Health Organization (WHO) estimated that in 2006, there were approximately 12 million new cases of syphilis [1], with the highest disease burden in sub-Saharan African and South and Southeast Asian countries where prenatal screening rates are often as low as 30% leading to high rates of stillbirths and congenital syphilis [1]. The Centers for Disease Control and Prevention (CDC) estimated that approximately 64% cases of syphilis in the United States of America (USA) were among men who have sex with men (MSM) [2]. Globally, lack of awareness concerning one's serostatus is one of the main driving forces of the syphilis epidemic as approximately 90% of those infected do not know they are infected [3]. Estimates of disease burden across global settings are under-estimated due to lack of regular screening initiatives for at risk populations, namely sexually transmitted diseases (STD) clinic attendees, MSM and pregnant women.

Syphilis is diagnosed using laboratory tests, consisting of non-*Treponema pallidum* (non-TP) and *Treponema pallidum* (TP) specific tests. First line screening is usually performed with non-TP tests that detect anti-cardiolipin antibodies such as rapid plasma reagin (RPR) and venereal diseases research laboratory (VDRL) tests that are popular globally [4]. These tests are cheaper compared to treponemal tests, but they detect a cardiolipin antigen that also occurs in many conditions other than syphilis such as auto-immune diseases and malaria. Therefore, confirming a preliminary positive RPR or VDRL with a treponemal specific test that detects antibodies to *Treponema pallidum* is necessary [4]. Some examples of typical treponemal specific tests are *Treponema pallidum* haemagglutination assay (TPHA), *Treponema pallidum* particle agglutination assay (TPAA) [4], and fluorescent treponemal antibody absorption (FTA-Abs) [5]. These tests are expensive, laboratory-based, require a continuous supply of electricity, reagents and trained staff, and are rarely available outside of reference laboratories [6]. As a result in many countries, treatment is based on RPR results leading to over-treatment of biologically false positive patients.

In recent years, syphilis rapid and point-of-care (POC) tests which detect antibodies to *T. pallidum* antigen have become popular due to their many advantages [6]. In order to define the ideal characteristics of a rapid and POC test, the WHO Sexually Transmitted Diseases Diagnostic Initiative (SDI) established the ASSURED criteria: Affordable, Sensitive, Specific, User-friendly, Rapid and robust, Equipment free, and Deliverable to those who need them [5], [7], [8]. Rapid and POC tests are performed on one patient at a time with results communicated to the patient within 20 minutes, saving time, preventing loss to follow up and allowing for same day treatment administration [6]. Rapid and POC tests thus offer the potential to increase access to screening, offering opportunities for Same-day Testing and Treatment (STAT) to prevent stillbirths or transmission of syphilis to the foetus if performed before the third trimester [1], [8]. However, treponemal antibodies persist for a long time, making it impossible to distinguish between active and past treated infection. In resource limited settings where most populations don't have access to a laboratory, those who are found to be seropositive by treponemal rapid and POC tests are treated for syphilis to interrupt potential transmission to a sexual partner or to the foetus in case of pregnant women. This is now accepted practice as the risk of over-treatment due to biological false positives which are not syphilis in origin is more acceptable than the risk of non-treatment of syphilis.

Despite these advantages, scepticism regarding accuracy of syphilis rapid and POC tests are an obstacle to widespread uptake. To date, only one review has evaluated the diagnostic accuracy of syphilis rapid and POC tests. This review only focused on specific sub-populations (i.e., antenatal clinic attendees and STD clinic attendees) [9]. Additionally, authors have avoided a head-to-head comparison of globally popular tests, and ignored the role of reference standards in assessing accuracy. A perfect reference standard for syphilis does not exist [5] and therefore, past data based on this approach are limited in their accuracy. To fill the current knowledge gap in accuracy of rapid and POC syphilis tests, a thorough comparative evaluation of all globally used rapid and POC tests using serum and blood samples was conducted. A Bayesian latent class analysis that does not assume availability of a perfect reference standard was used. This meta-analysis is important as it includes all global sub populations at risk, thus generating high quality evidence to inform global policy.

## Methods

The PRISMA guidelines were followed in conducting and reporting this meta-analysis [10].

### Search strategy

Five electronic databases (MEDLINE, EMBASE, GLOBAL HEALTH, BIOSIS, and Web of Science) were searched for the period of January 1980 to March 2012.

### Search string

The search string contained the following keywords: (syphilis OR *Treponema pallidum*) AND (Point-of-Care OR rapid test* OR rapid assay*). Articles reported in all languages and studies conducted involving human subjects were reviewed and translated.

### Study selection

**Figure 1:**
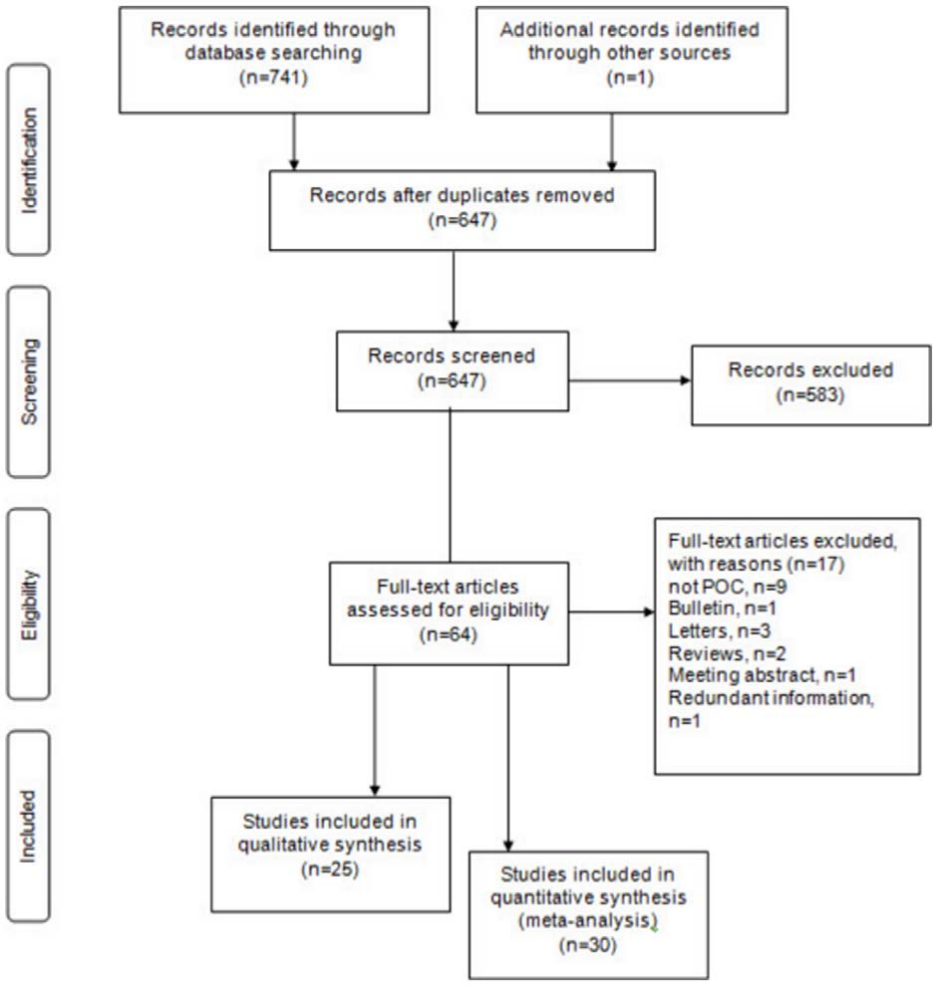
Study selection flow chart as per PRISMA guidelines.

Two reviewers (Yalda Jafari [YJ] and Sushmita Shivkumar [SS]) independently conducted the search. A third reviewer, (Nitika Pant Pai [NPP]), resolved discrepancies. Only studies that evaluated diagnostic accuracies and reported or allowed extraction of raw cell values (True Positive [TP], False Positive [FP], True Negative [TN], and False Negative [FN]) were included. Only rapid and POC tests that met the ASSURED criteria were included. Figure 1 depicts the study selection process.

### Data collection process

YJ extracted data from all included studies while SS independently extracted data from 30% of studies. Authors were contacted if further information was required.

Data items and summary measures: Information on author, year, location, population, study design, sample used, index test, reference test and raw cell values were extracted using a standardized data collection form. Outcomes were defined as sensitivity and specificity of the index test.

### Risk of bias in individual studies

Critical appraisal was undertaken using quality assessment of diagnostic accuracy studies (QUADAS) and standards for the reporting of diagnostic accuracy studies (STARD) respectively. QUADAS is a 14 item checklist used to evaluate the methodology of diagnostic studies [11] and STARD is a 25 item checklist used to evaluate the reporting of diagnostics studies [12].

### Synthesis of results

**Figure 2:**
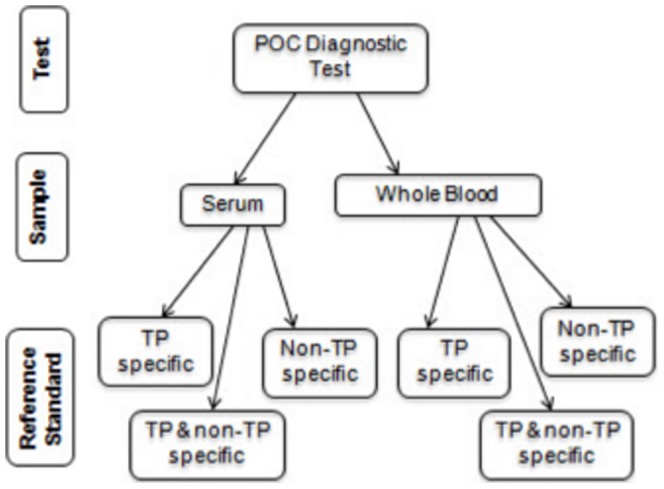
Stratification strategy employed to make subgroups for pooling.

Subgroups were created based on: a) index test, b) type of sample (serum or whole blood) and c) type of reference standards (TP specific, non-TP specific, or TP and non-TP specific). Biologically, results in serum are found to have higher sensitivity and specificity [9]. Therefore, it was important to consider the distinction between specimens that were tested. The distinction between reference tests (i.e., TP, non-TP, or TP and non-TP) was also considered important because results of each type of reference standard indicated different biological status and stage of infection. This assessment impacts the accuracy of the index test as the index tests are TP specific tests. Figure 2 summarizes the subgroup stratification strategy.

### Statistical analysis

Bayesian Hierarchical Summary Receiver Operating Characteristic Curve (HSROC) models were fit using the HSROC add-on package (version 2.0.5, Montreal) to R (version 2.12.1, 2010, The R Foundation for Statistical Computing). The program calculated overall sensitivities and specificities and estimated the SROC curve [13]. HSROC uses methods that allow for variability between index tests, studies, populations, and chance that impact evaluation of the accuracy of an index test [14]. In particular, it does not require reference standards to be considered as perfect indicators of true disease status, and there is a strong advantage of the latent class model used compared to other approaches. For reasonable estimation of all parameters, a minimum of four studies at the lowest stratification level is required.

### Model assumptions

Analyses were carried out assuming a perfect and imperfect reference standard. When no perfect reference standard is assumed, the statistical estimation problem becomes non-identifiable, meaning in practice that not all parameters can be estimated without the input of external information. Indeed, when a perfect reference standard is assumed, the external information is that the reference standard has 100% sensitivity and specificity. Using the methods implemented by the HSROC model, one can relax this almost surely false assumption by putting prior ranges on the likely values of the true sensitivity and specificity of the reference standard. For this purpose, prior distributions that were extracted from the literature were used. All priors were non-informative and the second level of the hierarchical model grouped the parameters (sensitivity and specificity) from each study.

### Assumptions of accuracy

Peeling et al. [5] reviewed conventional tests such as TPHA and TPPA and found sensitivities in the range of 85% to 100% and specificities in the range of 98% to 100%. RPGA, the reference standard used by Rotanov et al. [15], was classified as a test similar to TPHA and TPPA, and similar accuracy parameters were used. FTA-ABS was estimated to have a sensitivity of 70% to 100% and a specificity of 94% to 100% [5]. For combination TP and non-TP reference standard, an estimate of range of 90% to 100% for both sensitivity and specificity were assumed.

### Additional sensitivity analysis

The priors used to adjust for imperfect reference standards were derived from literature, and as such, they were evaluated against other imperfect reference standards. In view of this, a sensitivity analysis was conducted to determine the effect of priors on the estimates by widening the prior parameters used under the imperfect reference standard assumption, by 5%, 10% and 15% on each end of the range.

## Results

### Demographic characteristics

A list of included studies and their characteristics are presented in Table S1 x. Forty-four full text articles were assessed for eligibility, of which 33 (75%) were included in the meta-analysis. Some articles evaluated more than one index test, in more than one sample or more than one population, leading to 131 included data sets. A total of 58% (197/33) of articles evaluated the rapid and POC test using whole blood. In this group, 37% (7/19) of articles tested STD clinic attendees, 26% (5/19) tested female sex workers (FSW), 47% (9/19) tested antenatal clinic attendees (ANC) and 5% (1/19) used blood supplied by hospital. All studies were conducted on adults. In terms of settings, only about 10% (13/131) of studies were conducted in high-income countries. A majority (81%; 106/131) used a TP specific reference standard, 17% (22/131) used a combination reference standard and 2% (3/131) of studies used non-TP reference tests. For the purpose of this meta-analysis, articles were denoted as using adequate reference standards if the reference standard identified the same antibody as the index test.

### Characteristics of rapid and POC tests

Through this review, 18 rapid and POC tests in global use were identified. The vast majority were immuno-chromatographic strip (ICS) based assays with most tests being Determine (Abbott Diagnostics, UK) at 29% (38/131), SD Biolines (Standard, South Korea) at 18% (23/131), Syphicheck (Qualpro, India) at 15% (19/131) and VisiTect (Omega Diagnostics, UK) at 16% (21/131). A low number of studies employed non-TP and combination TP and non-TP reference standards, therefore, only meta-analysis of results using TP specific reference standard were found to be meaningful. As well, results of studies using of TP specific reference standard are biologically most valid as rapid and POC tests and TP specific reference standards both detect antibodies to *T. palladium* antigen.

### Meta-analysis output

Results of this meta-analysis are presented in Table 1 and the summary receiver operating characteristics (SROC) curves under the imperfect reference standard assumption are presented in Figures S1, S2, S3, S4, S5, S6, S7, S8, and S9.

**Table 1:** Results of pooled sensitivity and specificity, before and after adjustment for imperfect reference standard.

POC Test	Sample	Reference Standard	Pooled Parameters	Assuming Perfect Reference Standard (95% CrI)	Assuming Imperfect Reference Standard (95% CrI)	Reported Parameter by Manufacturer
Determine	Serum	TP Specific (n = 11)	Sensitivity	92.03% (87.22, 95.77)	90.04% (80.45, 95.21)	100%
			Specificity	92.68% (87.24, 95.87)	94.15% (89.26, 97.66)	100%
	Whole Blood	TP Specific (n = 15)	Sensitivity	79.54% (71.95, 86.04)	86.32% (77.26, 91.70)	92.30%
			Specificity	98.91% (97.71,99.71)	95.85% (92.42, 97.74)	100%
		TP & non-TP Specific (n = 8)	Sensitivity	77.68% (60.94, 90.40)	47.84% (18.25, 83.18)	NA
			Specificity	98.18% (94.42, 99.93)	97.81% (89.75, 99.79)	NA
SD Bioline	Serum	TP Specific (n = 8)	Sensitivity	87.88% (82.43, 91.80)	87.06% (75.67, 94.50)	99.3%
			Specificity	96.03% (92.67, 98.05)	95.85% (89.89, 99.53)	99.5%
	Whole Blood	TP Specific (n = 13)	Sensitivity	83.78% (80.89, 86.47)	84.50% (78.81, 92.61)	NA
			Specificity	98.39% (97.56, 99.01)	97.95% (92.54, 99.33)	NA
Syphicheck	Serum	TP Specific (n = 7)	Sensitivity	75.96% (63.55, 85.29)	74.48% (56.85, 88.44)	100%
			Specificity	98.51% (96.85, 99.59)	99.14% (96.37, 100.0)	NA
	Whole Blood	TP Specific (n = 12)	Sensitivity	75.12% (70.00,80.48)	74.47% (63.94, 82.13)	NA
			Specificity	99.44% (98.96, 99.81)	99.58% (98.91, 99.96)	NA
Visitect	Serum	TP Specific (n = 7)	Sensitivity	87.32% (79.97, 92.14)	85.13% (72.83, 92.57)	NA
			Specificity	95.76% (92.17, 97.76)	96.45% (91.92, 99.29)	NA
	Whole Blood	TP Specific (n = 14)	Sensitivity	76.22% (69.82, 81.62)	74.26% (53.62, 83.68)	NA
			Specificity	99.33% (98.73, 99.78)	99.43% (98.22, 99.98)	NA

CrI = Credible Interval; “n” refers to the number of data entries per group; NA = Not Available.

In whole blood sample, using a TP specific reference standard, under the imperfect reference standard assumption, sensitivity and specificity estimates with 95% credible intervals (CrI) for Determine was 86.32% (77.26, 91.70) and 95.85% (92.42, 97.74), for SD Bioline was 84.50% (78.81, 92.61) and 97.95% (92.54, 99.33), for Syphicheck was 74.47% (63.94, 82.13) and 99.58% (98.91, 99.96) and for VisiTect was 74.26% (53.62, 83.68) and 99.43% (98.22, 99.98).

The results of the sensitivity analysis are presented in Table 2. Generally, change in priors did not affect the accuracy. Except for sensitivity of Determine, in whole blood, using combination TP and non-TP specific reference standard which changed from around 45% to 77%, none of the changes were statistically significant.

**Table 2:** Results of sensitivity analysis.

POC Test	Sample	Reference Standard	Pooled Parameters	5% Widened Priors (95% CrI)*	10% Widened Priors (95% CrI)*	15% Widened Priors (95% CrI)*
Determine	Serum	TP Specific (n = 11)**	Sensitivity	90.11% (84.62, 94.48)	90.50% (84.70, 94.78)	88.89% (78.82, 96.25)
			Specificity	94.23% (90.07, 97.18)	93.97% (89.51, 97.26)	93.12% (85.21, 97.69)
	Whole Blood	TP Specific (n = 15)	Sensitivity	78.40% (69.72 85.50)	77.78% (67.66, 86.05)	78.23% (66.79, 87.21)
			Specificity	99.07% (97.92, 99.81)	99.01% (97.78, 99.77)	98.97% (97.10, 99.86)
		TP & non-TP Specific (n = 8)	Sensitivity	45.07% (16.10, 75.05)	62.35% (38.67, 85.64)	76.78% (58.80, 90.17)
			Specificity	98.83% (89.91, 99.98)	99.20% (96.24, 99.99)	98.48% (94.01, 99.98)
SD Bioline	Serum	TP Specific (n = 8)	Sensitivity	88.91% (81.06, 94.11)	88.57% (80.70, 93.88)	88.34% (80.01, 94.46)
			Specificity	94.77% (89.22, 98.72)	95.06% (90.12, 98.69)	95.17% (89.13, 98.83)
	Whole Blood	TP Specific (n = 13)	Sensitivity	77.45% (61.91, 87.52)	80.16% (58.30, 96.79)	82.60% (72.64, 88.70)
			Specificity	97.88% (94.74, 99.87)	97.90% (89.52, 99.98)	98.20% (96.61, 99.66)
Syphicheck	Serum	TP Specific (n = 7)	Sensitivity	61.14% (35.02, 82.86)	72.68% (50.60, 90.73)	71.45% (43.99, 91.00)
			Specificity	98.28% (92.36, 99.98)	98.99% (93.79, 100.0)	99.16% (94.42, 100.0)
	Whole Blood	TP Specific (n = 12)	Sensitivity	74.31% (62.03, 83.53)	74.56% (60.21, 83.11)	63.64% (45.56, 81.40)
			Specificity	99.54% (98.68, 99.99)	99.53% (98.68, 99.98)	99.20% (97.13, 99.98)
Visitect	Serum	TP Specific (n = 7)	Sensitivity	87.03% (77.00, 94.41)	85.46% (72.42, 93.92)	81.56% (64.86, 92.33)
			Specificity	95.64% (88.72, 99.15)	96.20% (90.32, 99.59)	95.63% (87.93, 99.35)
	Whole Blood	TP Specific (n = 14)	Sensitivity	73.10% (55.41, 84.13)	75.04% (57.52, 85.07)	76.34% (67.10, 83.98)
			Specificity	99.35% (97.58, 99.99)	99.41% (98.20, 99.98)	99.37% (98.40, 99.89)

CrI = Credible Interval; “n” refers to the number of data entries per group.

### Quality

Quality of methodology of studies was assessed using the QUADAS checklist. Results from the QUADAS evaluation of included articles are summarized in Table S2. There was minimal potential for incorporation bias (100%), all articles provided adequate description of execution of index test (100%) and reference tests (100%). The majority of articles used an adequate reference standard (97%). The majority also had absence of disease progression bias (97%) and absence of partial verification bias (94%). Potential for differential verification bias (91%) and selection bias (88%) was minimal. Five quality items were addressed in 60% of papers or less; absence of clinical review bias (45%), report of un-interpretable results (33%), absence of index test review bias (36%), absence of reference test review bias (24%), and description of withdrawals (21%). A limiting factor in assessing methodological quality was unclear reporting by authors. Absence of reference and index test review bias was unclear in 76% and 61% of articles respectively. Absence of clinical review bias was unclear in 51% of articles.

Quality of reporting of studies was assessed using the STARD checklist. These results are presented in Table S3. Only 4 of the 25 items were reported by all the articles. Of these, three items (items 7–9) were under methods; reference standard used and its rationale (100%), technical specifications of materials and methods (100%), definition of and rationale for units, and the clinical applicability of findings (100%). Items 5, 11, 13, 17, 20, 22, 23, and 24 were reported by 50% of articles or less, with item 20 (report of any adverse effects from use of index test or reference standard) being the least reported item (3%). About 33% (11/33) of papers reported on conflicts of interest, with each having at least one conflict of interest. In four articles [16]–[18], the authors evaluated the diagnostic test they had manufactured themselves or contributed to its manufacturing. The role of conflict of interest as a predictor of diagnostic performance could not be explored.

## Discussion

Using a TP specific reference standard in serum, the Determine rapid test had the best estimate for sensitivity and Syphicheck had the best specificity. Overall, rapid and POC tests performed well in both sensitivity and specificity compared to laboratory-based TP specific tests such as TPPA and TPHA that have sensitivity in the range of 85–100% and specificity in the range of 98–100% [5]. In this meta-analysis, correcting for imperfect reference standards improved the accuracy estimates of all rapid and POC tests. In all comparisons, estimates in serum were higher than estimates in whole blood. This was expected because of high concentration of biomarkers in serum and the absence of interfering substances in whole blood. It should be noted that meta-analysis results from the whole blood group assuming an imperfect TP specific reference standard are most relevant to decision makers as they best capture circumstances most encountered in resource constrained settings.

There are limitations to the use of rapid and POC syphilis tests that arise from the inherent characteristics of the tests. The rapid and POC tests analyzed in this review are treponemal tests and thus detect antibodies to *T. pallidum*. As previously mentioned, treponemal tests cannot be used to distinguish between active and past infection as antibodies to *T. pallidum* persist even if the patient is successfully treated [6]. In resource limited settings, these tests have been shown to be useful in detecting infection in patients with no previous access to testing or at risk populations marginalised from care or unlikely to return for results, and where failure in timely treatment can have adverse impacts on disease progression and transmission [5], [6], [8].

In a study conducted in Brazil, Mabey et al. [8] found rapid and POC tests to be helpful in the Amazonas region, a remote location where no testing was previously possible. In the case of syphilis, rapid and POC tests can easily facilitate initiation of treatment and prevention of transmission and reduce the reservoir of infection in the community. Following a positive result with a rapid and POC test, a first dose of penicillin is recommended to be administered on the same day [8]. In pregnant women, a single dose of penicillin before the third trimester is adequate to prevent neonatal deaths and stillbirths [8]. Watson-Jones et al. [19] found that the rate of adverse pregnancy outcome is similar in pregnant women treated with single-dose penicillin before the end of the second trimester compared to those seronegative for syphilis. Considering the adverse outcomes associated with maternal syphilis as well as chances of onward transmission to partners, the benefit of screening using rapid and POC tests far outweighs the risk of a small proportion of missed cases due to false negative results or over-treatment due to a past infection in resource limited settings..

Improvements in accuracy parameters that reduce the occurrence of false positives and false negatives as well as addition of non-TP components to rapid and POC tests are warranted, but waiting for a “perfect test” will mean that over 500,000 babies could be stillborn or die of congenital syphilis every year [8]. Three studies and models have clearly demonstrated a positive impact of using these less than perfect rapid and POC tests on reducing the disease burden of syphilis in resource limited settings.

Aledort et al. [20] described a model, funded by the Bill & Melinda Gates Foundation, that showed that a diagnostic test for antenatal syphilis screening that meets the performance of the current laboratory-based RPR test (86% sensitivity and 72% specificity), but requires no infrastructure, and assuming a 100% treatment rate, would save more than 201,000 lives and avert 215,000 stillbirths. In the model, the positive outcomes of using such a test are more dependent on effective diagnoses and treatment rather than inappropriate treatment, as treatment has generally low harm [20]. Penicillin has rarely any side effects when given at the therapeutic doses [21], [22], there is no evidence that *T. pallidum* has developed resistance to penicillin [5], and it is inexpensive [23]. The rapid and POC tests in this meta-analysis fulfilled the performance threshold in the model and can hence be recommended to save lives in resource limited settings. In another study, conducted by Vickerman et al. [24], potential benefits of rapid and POC tests instead of RPR were modeled. Results showed that rapid and POC tests led to a higher treatment rate than RPR. In a study by Bronzan et al. [25] in rural South Africa, rapid and POC tests, in comparison to other methods, resulted in the highest percentage of pregnant women correctly diagnosed and treated for syphilis (89.4% for rapid tests, 63.9% for on-site RPR, 60.8% for offsite RPR/TPHA). The on-site RPR had low sensitivity (71.4% for high-titer syphilis) while the offsite approach suffered from poor client return rates. One percent of women screened with the rapid and POC tests may have received penicillin unnecessarily but there were no adverse treatment outcomes [25]. These studies serve to show that rapid and POC tests can reduce syphilis-related adverse outcomes of pregnancy through timely diagnosis and immediate treatment of pregnant women with syphilis.

In this meta-analysis, the POC tests evaluated are TP –specific tests and as such, they were compared to TP-specific reference tests. The goal was to determine their accuracy as a screening tool, which in an ideal setting, will be used in an algorithm with other tests such as non-TP specific tests to diagnose syphilis. Non-TP antibodies are generally a better indicator of active syphilis infection as the antibodies decline after treatment. However, non-TP tests measure anti-cardiolipin antibodies, which can give rise to false positive due to presence of factors such as malaria, immune disorders, and pregnancy.

In Watson-Jones's study from Tanzania, as many as 27.5% of RPR positive results in pregnant women could not be confirmed by the *Treponema pallidum* Particle Agglutination (TPPA) assay [19]. In addition, Creegan et al. found that non-TP tests were less sensitive than TP tests in detecting early syphilis [26].

This meta-analysis is concerned with the use of these rapid POC tests in resource limited settings where laboratory infrastructures are limited or non-existent. In 2004, Schmid et al. reported that in developing countries, an estimated 68% of urban women and 39% of rural women access antenatal care [27]. Universal institution of an effective programme for syphilis screening and treating those who are seropositive would avoid 492,000 deaths in Africa alone. Screening rates remain very low in most rural settings. However, rapid and POC tests, despite their imperfect performance, have already been shown in large implementation pilot studies to have averted many deaths [8].

A great step forward is the new rapid and POC tests that simultaneously detect TP and non TP specific antibodies such as the assay evaluated by Castro et al. [16], [28]. Such tests provide a clear advantage over tests that only detect TP antibodies by helping to distinguish between past and current infections. However, as of this year, there was limited published data in this regard to allow for exploration of their accuracy. More research on these combination tests is highly warranted. These tests will certainly help expedite screening and referral for treatment in the years to come.

This is the first meta-analysis providing a comparison of the diagnostic accuracy of popular rapid and POC tests for syphilis. In a previous review conducted by Tucker et al. [9], data were analyzed using a standard meta-analysis method that does not adjust for the imperfections of reference standards. In this review, adjustments were made for imperfect reference standards using Bayesian HSROC method, literature were explored in all languages, and quality of evidence was rated with validated checklists. The accuracy of rapid and POC syphilis tests was estimated by examining the global evidence and analyzing results by indivdual tests, sample and reference standards, thus prodviding evidence useful for policy and planning. Additionally, implementation research outcomes such as acceptability, feasibility and economic outcomes were synthesized in a separate review by the authors of this article [29].

### Limitations

This meta-analysis is subject to limitations that should be borne in mind while interpreting results. Although a comprehensive search strategy was implemented, it is possible that relevant studies may have been missed, thus there is potential for publication bias. There is also a possibility that studies that showed higher accuracy are published more favorably. However, exploration of publication bias with funnel plots is not recommended in meta-analyses and was not carried out [14]. As well, the reviewers were un-blinded to the authors and institutions that may potentially introduce detection bias [12].

There are limitations to the statistical analysis conducted. Prior distributions used for the HSROC analysis assuming imperfect reference standard were obtained from literature and themselves measured against other TP and non-TP specific tests. As a result, there is no guarantee that these ranges are correct. Hence, interpretations of Bayesian inferences must be made conditional on choice of priors. Thus, the analysis was limited by the current methodology of diagnostic accuracy meta-analyses.

Moreover, lack of data limited further analysis. There is a visible time trend in this review, with studies spanning almost two decades. There have been many improvements in conjugates and test devices during this period. However, with limitations in data from various settings and type of devices, it was not possible to sort the data by years and evaluate the trajectory of improving diagnostic accuracy over time. Lack of data also did not allow for further stratification in order to investigate the effect of using various samples, such as whole blood obtained from fingerstick or venipuncture which is treated with anti-coagulants. Exploration of accuracy of rapid and POC tests by stage of infection and status of co-infections was hindered by lack of available data. Future studies may need to explore the accuracy of rapid and POC tests by stage and by co-infection status in greater detail, particularly in the light of integrated multiplexed screening initiatives for several STIs that are under development. Due to lack of data availability, analysis was largely generated using treponemal tests, which overestimate sensitivity of detecting acute, untreated disease [30].

There were limitations due to poor methodology and reporting in the publications. A low number of studies (33%) reported on conflicts of interest and only 24% reported on whether the study was conducted blindly, raising the possibility of detection bias. Journals should promote greater standards of reporting in order to promote transparency. Barring these caveats, popular rapid and POC tests were compared in various samples to obtain an assessment of their global accuracy to inform evidence based decision making for policy initiatives.

### Conclusion

This meta-analysis found that rapid and POC treponemal tests had high sensitivity and specificity in serum and blood that was at par or better than laboratory-based non-treponemal tests currently in use in most resource limited settings. Despite being less than 100% accurate, in areas where there is no access to laboratory screening facilities for syphilis, rapid and POC tests have the potential to facilitate rapid improved detection of syphilis, allowing for treatment initiation in the same visit, reducing missed opportunities for detection and timely intervention to prevent transmission to infants and to partners. Based on the evidence, it is concluded that rapid and POC tests are useful in global settings with limited access to laboratories or screening for syphilis. The duplex rapid TP- non-TP rapid and POC test as well as improvements in sensitivity will prevent over diagnosis and over treatment of syphilis in these settings.

## Supporting Information

Table S1 **Characteristics and results of studies included in Part I.**
(DOC)

Table S2 **Results of QUADAS evaluation.**
(DOC)

Table S3 **Results of STARD evaluation.**
(DOC)

Figure S1 **Summary ROC curve for Determine, in serum, using imperfect TP specific reference standard.**
(TIF)

Figure S2 **Summary ROC curve for Determine, in whole blood, using imperfect TP specific reference standard.**
(TIF)

Figure S3 **Summary ROC curve for Determine, in whole blood, using imperfect TP and non-TP specific reference standard.**
(TIF)

Figure S4 **Summary ROC curve for SD Bioline, in serum, using imperfect TP specific reference standard.**
(TIF)

Figure S5 **Summary ROC curve for SD Bioline, in whole blood, using imperfect TP specific references standard.**
(TIF)

Figure S6 **Summary ROC curve for Syphicheck, in serum, using imperfect TP specific reference standard.**
(TIF)

Figure S7 **Summary ROC curve for Syphicheck, in whole blood, using imperfect TP specific reference standard.**
(TIF)

Figure S8 **Summary ROC curve for Visitect, in serum, using imperfect TP specific reference standard.**
(TIF)

Figure S9 **Summary ROC curve for Visitect, in whole blood, using imperfect TP specific reference standard.**
(TIF)
