# Supplementary material for: Are Treponema pallidum Specific Rapid and Point-of-Care Tests for Syphilis Accurate Enough for Screening in Resource Limited Settings? Evidence from a Meta-Analysis
Source: PLoS One. 2013 Feb 26;8(2):e54695. doi: 10.1371/journal.pone.0054695 (PMC3582640; doi:10.1371/journal.pone.0054695)
Supplement: Table S1 — Characteristics and results of studies included in Part I. (DOC) [file pone.0054695.s001.doc]

Table 1: Characteristics and results of studies included in Part I.

| **Study  ID** | **Author** | **Year** | **Location** | **Study Design** | **Reference Standard** | **Index Test** | **Sample** | **TP** | **FN** | **FP** | **TN** | **Population** |
| --- | --- | --- | --- | --- | --- | --- | --- | --- | --- | --- | --- | --- |
| 1 | Benzaken | 2007 | Manaus, Brazil (UMI) | Cross-sectional | FTA-Abs | SD Bioline | Whole Blood | 45 | 6 | 3 | 487 | STD clinic attendees |
| 1 | Benzaken | 2007 | Manaus, Brazil (UMI) | Cross-sectional | FTA-Abs | SD Bioline | Serum | 46 | 5 | 3 | 487 | STD clinic attendees |
| 1 | Benzaken | 2007 | Manaus, Brazil (UMI) | Cross-sectional | FTA-Abs | Syphicheck | Whole Blood | 43 | 8 | 2 | 488 | STD clinic attendees |
| 1 | Benzaken | 2007 | Manaus, Brazil (UMI) | Cross-sectional | FTA-Abs | Syphicheck- | Serum | 45 | 6 | 2 | 488 | STD clinic attendees |
| 1 | Benzaken | 2007 | Manaus, Brazil (UMI) | Cross-sectional | FTA-Abs | VisiTect | Whole Blood | 50 | 2 | 3 | 193 | STD clinic attendees |
| 1 | Benzaken | 2007 | Manaus, Brazil (UMI) | Cross-sectional | FTA-Abs | VisiTect | Serum | 50 | 2 | 3 | 193 | STD clinic attendees |
| 1 | Benzaken | 2007 | Manaus, Brazil (UMI) | Cross-sectional | FTA-Abs | Determine | Whole Blood | 46 | 6 | 4 | 192 | STD clinic attendees |
| 1 | Benzaken | 2007 | Manaus, Brazil (UMI) | Cross-sectional | FTA-Abs | Determine | Serum | 46 | 6 | 4 | 192 | STD clinic attendees |
| 2 | Benzaken | 2008 | Manaus, Brazil (UMI) | Cross-sectional | FTA-Abs | VisiTect | Whole Blood | 52 | 40 | 5 | 409 | Outreach clinic |
| 3 | Benzaken | 2011 | Alto Solimoes, Brazil UMI) | Cross-sectional | FTA-Abs | VisiTect | Whole Blood | 10 | 6 | 6 | 690 | ANC attendees |
| 4 | Bronzan | 2007 | Cape town,  South Africa(UMI) | Cross-sectional | RPR + TPHA | Determine | Whole Blood | 20 | 2 | 29 | 290 | ANC attendees |
| 5 | Campos(a) | 2006 | Lima, Peru (UMI) | Cross-sectional | RPR reactivity at any dilution  + TPHA | Determine | Whole Blood | 70 | 108 | 28 | 3277 | FSWs in comercial sex venues |
| 5 | Campos(b) | 2006 | Lima, Peru (UMI) | Cross-sectional | RPR >=1:8 + TPHA | Determine | Whole Blood | 16 | 9 | 82 | 3376 | FSWs in comercial sex venues |
| 5 | Campos(c) | 2006 | Lima, Peru (UMI) | Cross-sectional | RPR>=1:16 + TPHA | Determine | Whole Blood | 7 | 3 | 91 | 3382 | FSWs in comercial sex venues |
| 6 | Castro | 2010 | Atlanta, United States (HI) | Case-Control | TPPA | Immunofiltration device(Span diagnositcs ltd, Surat, India) | Serum | 147 | 4 | 2 | 223 | Archived serum samples |
| 7 | Castro | 2010 | Atlanta, United States (HI) | Case-Control | TPPA | Immunochromatographic device*(Chembio Diagnostics Systems Inc.Medford, NY) | Serum | 972 | 35 | 27 | 567 | Archived serum samples |
| 8 | Diaz(a) | 2004 | Rio de Janeiro, Brazil (UMI) | Cross-sectional | TPHA | Determine | Serum | 244 | 6 | 8 | 292 | Infectious disease research attendees |
| 8 | Diaz(b) | 2004 | Rio de Janeiro, Brazil (UMI) | Cross-sectional | TPHA | Determine | Serum | 246 | 4 | 13 | 287 | Infectious disease research attendees |
| 8 | Diaz(c) | 2004 | Rio de Janeiro, Brazil (UMI) | Cross-sectional | TPHA | Determine | Serum | 241 | 9 | 11 | 289 | Infectious disease research attendees |
| 9 | Gianino | 2007 | Turin, Italy (HI) | Cross-sectional | Clinical symptoms + ELISA + RPR  or TP-PA or ELISA IgM | Determine | Whole Blood | 94 | 5 | 5 | 212 | High Risk |
| 10 | Hernandez-Trejo | 2006 | Cuernavaca City and Mexico City, Mexico ((UMI) | Cross-sectional | VDRL + FTA-Abs | Determine | Whole Blood | 4 | 0 | 0 | 1318 | Pregnant Women |
| 11 | Huang(a) | 1996 | China (LMI) | Case-Control | RPR | Dot-Immunogold  Filtration Assay (DIGFA) | Serum | 50 | 0 | 0 | 300 | Clinical Samples |
| 11 | Huang(b) | 1996 | China (LMI) | Case-Control | FTA-ABS | Dot-Immunogold  Filtration Assay (DIGFA) | Serum | 48 | 0 | 2 | 300 | Clinical Samples |
| 12 | Juarez-Figueroa(a) | 2007 | Cuernavaca City, Mexico (UMI), | Cross-sectional | VDRL + FTA-Abs | Determine | Serum | 57 | 1 | 4 | 78 | FSWs attending urban clinic |
| 12 | Juarez-Figueroa(b) | 2007 | Mexico City, Mexico (UMI) | Cross-sectional | VDRL + FTA-Abs | Determine | Whole Blood | 30 | 1 | 0 | 167 | FSWs attending STI clinic |
| 12 | Juarez-Figueroa(c) | 2007 | Cuernavaca City, Mexico (UMI) | Cross-sectional | VDRL + FTA-Abs | Determine | Whole Blood | 3 | 0 | 1 | 196 | Pregnant Women attending urban clinic |
| 13 | Li(a) | 2009 | Beijing, China (LMI) | Cross-sectional | TPHA | Determine | Whole Blood | 68 | 15 | 2 | 360 | STD clinic attendees |
| 13 | Li(b) | 2009 | Beijing, China  (LMI) | Cross-sectional | TPHA | Determine | Whole Blood | 64 | 19 | 0 | 362 | STD clinic attendees |
| 13 | Li(c) | 2009 | Beijing, China  (LMI) | Cross-sectional | TPHA | Determine | Serum | 83 | 0 | 4 | 358 | STD clinic attendees |
| 13 | Li(d) | 2009 | Beijing, China  (LMI) | Cross-sectional | TPHA | VisiTect | Whole Blood | 61 | 22 | 1 | 361 | STD clinic attendees |
| 13 | Li(e) | 2009 | Beijing, China  (LMI) | Cross-sectional | TPHA | VisiTect | Whole Blood | 64 | 19 | 0 | 362 | STD clinic attendees |
| 13 | Li(f) | 2009 | Beijing, China  (LMI) | Cross-sectional | TPHA | VisiTect | Serum | 78 | 5 | 7 | 362 | STD clinic attendees |
| 13 | Li(g) | 2009 | Beijing, China (LMI) | Cross-sectional | TPHA | Syphicheck | Whole Blood | 57 | 32 | 1 | 325 | STD clinic attendees |
| 13 | Li(h) | 2009 | Beijing, China (LMI) | Cross-sectional | TPHA | Syphicheck | Whole Blood | 63 | 26 | 1 | 325 | STD clinic attendees |
| 13 | Li(i) | 2009 | Beijing, China (LMI) | Cross-sectional | TPHA | Syphicheck | Serum | 60 | 29 | 4 | 322 | STD clinic attendees |
| 13 | Li(j) | 2009 | Beijing, China (LMI) | Cross-sectional | TPHA | SD Bioloine | Whole Blood | 78 | 11 | 1 | 325 | STD clinic attendees |
| 13 | Li(k) | 2009 | Beijing, China (LMI) | Cross-sectional | TPHA | SD Bioline | Whole Blood | 78 | 11 | 1 | 325 | STD clinic attendees |
| 13 | Li(l) | 2009 | Beijing, China (LMI) | Cross-sectional | TPHA | SD Bioline | Serum | 85 | 4 | 7 | 319 | STD clinic attendees |
| 14 | Lien | 2000 | Ho Chi Minh City, Vietnam (LMI) | Cross-sectional | VDRL Carbon + Antigen RPR | Determine | Serum | 72 | 0 | 3 | 216 | potential cross  reactives |
| 15 | Mabey(a) | 2006 | Haiti (LI) | Cross-sectional | TPHA | Determine | Whole Blood | 29 | 11 | 11 | 710 | STI clinic attendees |
| 15 | Mabey(b) | 2006 | Haiti (LI) | Cross-sectional | TPHA | Determine | Whole Blood | 40 | 0 | 31 | 690 | STI clinic attendees |
| 15 | Mabey(c) | 2006 | Haiti (LI) | Cross-sectional | TPHA | Determine | Serum | 40 | 0 | 31 | 690 | STI clinic attendees |
| 15 | Mabey(d) | 2006 | China (LMI) | Cross-sectional | TPHA | Determine | Whole Blood | 68 | 15 | 2 | 360 | STI clinic attendees |
| 15 | Mabey(e) | 2006 | China (LMI) | Cross-sectional | TPHA | Determine | Whole Blood | 64 | 19 | 0 | 362 | STI clinic attendees |
| 15 | Mabey(f) | 2006 | China (LMI) | Cross-sectional | TPHA | Determine | Serum | 83 | 0 | 4 | 358 | STI clinic attendees |
| 15 | Mabey(g) | 2006 | Tanzania (LI) | Cross-sectional | TPHA | Determine | Whole Blood | 34 | 23 | 3 | 468 | ANC attendees |
| 15 | Mabey(h) | 2006 | Tanzania (LI) | Cross-sectional | TPHA | Determine | Whole Blood | 46 | 11 | 3 | 468 | ANC attendees |
| 15 | Mabey(i) | 2006 | Tanzania (LI) | Cross-sectional | TPHA | Determine | Serum | 52 | 5 | 10 | 461 | ANC attendees |
| 15 | Mabey(j) | 2006 | Brazil (UMI) | Cross-sectional | TPHA | Determine | Whole Blood | 46 | 8 | 4 | 191 | STI clinic attendees |
| 15 | Mabey(k) | 2006 | Brazil (UMI) | Cross-sectional | TPHA | Determine | Serum | 46 | 8 | 4 | 191 | STI clinic attendees |
| 15 | Mabey(l) | 2006 | Haiti (LI) | Cross-sectional | TPHA | VisiTect | Whole Blood | 40 | 15 | 4 | 457 | STI clinic attendees |
| 15 | Mabey(m) | 2006 | Haiti (LI) | Cross-sectional | TPHA | VisiTect | Whole Blood | 54 | 4 | 6 | 455 | STI clinic attendees |
| 15 | Mabey(n) | 2006 | Haiti (LI) | Cross-sectional | TPHA | VisiTect | Serum | 54 | 1 | 6 | 455 | STI clinic attendees |
| 15 | Mabey(o) | 2006 | China(LMI) | Cross-sectional | TPHA | VisiTect | Whole Blood | 61 | 22 | 1 | 361 | STI clinic attendees |
| 15 | Mabey(p) | 2006 | China(LMI) | Cross-sectional | TPHA | VisiTect | Whole Blood | 63 | 20 | 0 | 362 | STI clinic attendees |
| 15 | Mabey(q) | 2006 | China (LMI) | Cross-sectional | TPHA | VisiTect | Serum | 78 | 5 | 7 | 355 | STI clinic attendees |
| 15 | Mabey(r) | 2006 | Tanzania (LI) | Cross-sectional | TPHA | VisiTect | Whole Blood | 43 | 12 | 5 | 522 | ANC attendees |
| 15 | Mabey(s) | 2006 | Tanzania (LI) | Cross-sectional | TPHA | VisiTect | Whole Blood | 46 | 11 | 2 | 469 | ANC attendees |
| 15 | Mabey(t) | 2006 | Tanzania (LI) | Cross-sectional | TPHA | VisiTect | Serum | 48 | 9 | 4 | 467 | ANC attendees |
| 15 | Mabey(u) | 2006 | Brazil (UMI) | Cross-sectional | TPHA | VisiTect | Whole Blood | 49 | 2 | 3 | 190 | STI clinic attendees |
| 15 | Mabey(v) | 2006 | Brazil (UMI) | Cross-sectional | TPHA | VisiTect | Serum | 49 | 2 | 3 | 190 | STI clinic attendees |
| 15 | Mabey(w) | 2006 | Haiti (LI) | Cross-sectional | TPHA | Syphicheck | Whole Blood | 33 | 8 | 11 | 491 | STI clinic attendees |
| 15 | Mabey(x) | 2006 | Haiti (LI) | Cross-sectional | TPHA | Syphicheck | Whole Blood | 40 | 1 | 7 | 495 | STI clinic attendees |
| 15 | Mabey(y) | 2006 | Haiti (LI) | Cross-sectional | TPHA | Syphicheck | Serum | 40 | 1 | 8 | 494 | STI clinic attendees |
| 15 | Mabey(z) | 2006 | China (LMI) | Cross-sectional | TPHA | Syphicheck | Whole Blood | 57 | 32 | 1 | 325 | STI clinic attendees |
| 15 | Mabey(aa) | 2006 | China (LMI) | Cross-sectional | TPHA | Syphicheck | Whole Blood | 63 | 26 | 1 | 325 | STI clinic attendees |
| 15 | Mabey(bb) | 2006 | China (LMI) | Cross-sectional | TPHA | Syphicheck | Serum | 60 | 29 | 4 | 322 | STI clinic attendees |
| 15 | Mabey(cc) | 2006 | Tanzania (LI) | Cross-sectional | TPHA | Syphicheck | Whole Blood | 43 | 12 | 5 | 522 | ANC attendees |
| 15 | Mabey(dd) | 2006 | Tanzania (LI) | Cross-sectional | TPHA | Syphicheck | Whole Blood | 47 | 8 | 5 | 522 | ANC attendees |
| 15 | Mabey(ee) | 2006 | Tanzania (LI) | Cross-sectional | TPHA | Syphicheck | Serum | 48 | 7 | 6 | 521 | ANC attendees |
| 15 | Mabey(ff) | 2006 | Brazil (UMI) | Cross-sectional | TPHA | Syphicheck | Whole Blood | 42 | 8 | 2 | 490 | FSWs |
| 15 | Mabey(gg) | 2006 | Brazil (UMI) | Cross-sectional | TPHA | Syphicheck | Serum | 44 | 6 | 2 | 490 | STI clinic attendees |
| 15 | Mabey(hh) | 2006 | Haiti (LI) | Cross-sectional | TPHA | SD Bioline | Whole Blood | 30 | 0 | 8 | 477 | STI clinic attendees |
| 15 | Mabey(ii) | 2006 | Haiti (LI) | Cross-sectional | TPHA | SD Bioline | Whole Blood | 29 | 1 | 7 | 478 | STI clinic attendees |
| 15 | Mabey(jj) | 2006 | Haiti (LI) | Cross-sectional | TPHA | SD Bioline | Serum | 30 | 0 | 7 | 478 | STI clinic attendees |
| 15 | Mabey(kk) | 2006 | China (LMI) | Cross-sectional | TPHA | SD Bioline | Whole Blood | 78 | 11 | 2 | 324 | STI clinic attendees |
| 15 | Mabey(ll) | 2006 | China (LMI) | Cross-sectional | TPHA | SD Bioline | Whole Blood | 78 | 11 | 2 | 324 | STI clinic attendees |
| 15 | Mabey(mm) | 2006 | China (LMI) | Cross-sectional | TPHA | SD Bioline | Serum | 85 | 4 | 7 | 319 | STI clinic attendees |
| 15 | Mabey(nn) | 2006 | Tanzania (LI) | Cross-sectional | TPHA | SD Bioline | Whole Blood | 57 | 9 | 10 | 506 | ANC attendees |
| 15 | Mabey(oo) | 2006 | Tanzania (LI) | Cross-sectional | TPHA | SD Bioline | Whole Blood | 60 | 6 | 20 | 496 | ANC attendees |
| 15 | Mabey(pp) | 2006 | Tanzania (LI) | Cross-sectional | TPHA | SD Bioline | Serum | 60 | 6 | 23 | 493 | ANC attendees |
| 15 | Mabey(qq) | 2006 | Brazil (UMI) | Cross-sectional | TPHA | SD Bioline | Whole Blood | 44 | 6 | 3 | 489 | STI clinic attendees |
| 15 | Mabey(rr) | 2006 | Brazil (UMI) | Cross-sectional | TPHA | SD Bioline | Serum | 45 | 5 | 3 | 489 | STI clinic attendees |
| 16 | Miranda | 2009 | Brazil (UMI) | Cross-Sectional | TPHA | Determine | Whole Blood | 4 | 1 | 2 | 1373 | Antenatal clinic attendants in Labor |
| 17 | Mishra(a) | 2010 | Bangalore, India (LMI) | Cross-sectional | TPHA | Biorapid Syphilis | Whole Blood | 114 | 128 | 4 | 1371 | FSWs |
| 17 | Mishra(b) | 2010 | Bangalore, India (LMI) | Cross-sectional | RPR + TPHA | Biorapid Syphilis | Whole Blood | 85 | 35 | 33 | 1464 | FSWs |
| 17 | Mishra(c) | 2010 | Bangalore, India (LMI) | Cross-sectional | RPR (titre>/1:8) + TPHA | Biorapid Syphilis | Whole Blood | 44 | 16 | 74 | 1483 | FSWs |
| 17 | Mishra(d) | 2010 | Bangalore, India (LMI) | Cross-sectional | TPHA | Biorapid Syphilis | Serum | 193 | 49 | 19 | 1356 | FSWs |
| 17 | Mishra(e) | 2010 | Bangalore, India (LMI) | Cross-sectional | RPR + TPHA | Biorapid Syphilis | Serum | 117 | 3 | 95 | 1402 | FSWs |
| 17 | Mishra(f) | 2010 | Bangalore, India (LMI) | Cross-sectional | RPR (titre>/1:8) + TPHA | Biorapid Syphilis | Serum | 60 | 0 | 152 | 1405 | FSWs |
| 18 | Montoya(a) | 2006 | Sofala province, Mozambique (LI) | Cross-sectional | TPHA | SD Bioline | Serum | 488 | 44 | 25 | 3912 | Pregnant Women |
| 18 | Montoya(b) | 2006 | Sofala province, Mozambique (LI) | Cross-sectional | TPHA + RPR | SD Bioline | Serum | 367 | 15 | 146 | 3941 | Pregnant Women |
| 18 | Montoya(c) | 2006 | Sofala province, Mozambique (LI) | Cross-sectional | TPHA | SD Bioline | Whole Blood | 420 | 111 | 39 | 3892 | Pregnant Women |
| 18 | Montoya(d) | 2006 | Sofala province, Mozambique (LI) | Cross-sectional | TPHA + RPR | SD Bioline | Whole Blood | 327 | 54 | 132 | 3949 | Pregnant Women |
| 19 | Nessa(a) | 2008 | Mirpur, Dhaka, Bangladesh (LI) | Cross-sectional | RPR+TPHA | ICS-ACON | Whole Blood | 138 | 4 | 43 | 499 | FSW |
| 19 | Nessa(b) | 2008 | Mirpur, Dhaka, Bangladesh (LI) | Cross-sectional | RPR+TPHA | ICS-ACON | Whole Blood | 134 | 8 | 40 | 502 | FSW |
| 19 | Nessa(c) | 2008 | Mirpur, Dhaka, Bangladesh (LI) | Cross-sectional | RPR+TPHA | RTD-ACON | Whole Blood | 135 | 7 | 38 | 504 | FSW |
| 19 | Nessa(d) | 2008 | Mirpur, Dhaka, Bangladesh (LI) | Cross-sectional | RPR+TPHA | RTD-ACON | Whole Blood | 123 | 19 | 38 | 504 | FSW |
| 20 | Nyamwamu(a) | 2009 | Eldoret, Kenya (LI) | Cross-sectional | VDRL | Accurate Ultra Rapid | Serum | 4 | 1 | 2 | 143 | ANC attendees |
| 20 | Nyamwamu(b) | 2009 | Eldoret, Kenya (LI) | Cross-sectional | TPHA | Accurate Ultra Rapid | Serum | 6 | 0 | 0 | 144 | ANC attendees |
| 21 | Oshiro(a) | 1999 | Japan (HI) | Case-Control | Mediace TPLA | DainaScreen | Serum | 67 | 0 | 0 | 69 | Commercil panel and clinical Samples |
| 21 | Oshiro(b) | 1999 | Japan (HI) | Case-Control | FTA-ABS | DainaScreen | Serum | 34 | 0 | 0 | 66 | Commercil panel and clinical Samples |
| 21 | Oshiro(c) | 1999 | Japan (HI) | Case-Control | FTA-ABS | DainaScreen | Whole Blood | 34 | 0 | 0 | 66 | Commercil panel and clinical Samples |
| 22 | Rotanov(a) | 2008 | Russia (UMI) | Case-Control | RPGA | Determine | serum | 50 | 0 | 0 | 50 | Archived serum panels |
| 22 | Rotanov(b) | 2008 | Russia (UMI) | Case-Control | RPGA | Bioline Syphilis anti-TP Test Card | serum | 48 | 1 | 2 | 49 | Archived serum panels |
| 22 | Rotanov(c) | 2008 | Russia (UMI) | Case-Control | RPGA | Treponema-Express | serum | 50 | 5 | 0 | 45 | Archived serum panels |
| 23 | Rotty | 20 | Melbourne, Australia (HI) | Case-control | EIA-IgM | IgM Assay | Serum | 14 | 5 | 3 | 101 | Archived serum panels |
| 24 | Sano | 1999 | Japan (HI) | Case-Control | TPPA | DainaScreen | Serum | 0 | 0 | 3 | 997 | Clinical Samples |
| 25 | Sato | 2003 | Sao Paulo, SP, Brazil (UMI) | Case-Control | Symptomatic diagnosis | Determine | Serum | 59 | 4 | 3 | 59 | STD clinic attendees |
| 26 | Siedner(a) | 2004 | San Francisco, USA (HI) | Crpss-Sectional | TP-PA | Determine | Whole Blood | 52 | 0 | 0 | 47 | City Clinic |
| 26 | Siedner(b) | 2004 | San Francisco, USA (HI) | Cross-Sectional | TPPA | Determine | Whole Blood | 60 | 8 | 0 | 59 | City Clinic |
| 26 | Siedner(c) | 2004 | San Francisco, USA (HI) | Cross-Sectional | TPPA | Phoenix Biotech  Trep-Strip IV | Whole Blood | 23 | 10 | 0 | 38 | City Clinic |
| 26 | Siedner(d) | 2004 | San Francisco, USA (HI) | Cross-Sectional | TPPA | Guardian One Step | Whole Blood | 41 | 16 | 0 | 59 | City Clinic |
| 27 | Tinajeros | 2006 | Bolivia (LMI) | Cross-sectional | RPR + TPPA | Determine | Whole Blood | 314 | 28 | 128 | 8422 | Pregnant Women |
| 28 | van Dommelen | 2008 | Maastricht, the Netherlands (HI) | Case-Control | TPPA | Biorapid Syphilis | Serum | 133 | 12 | 39 | 146 | University Hospital |
| 29 | Villazon-Vargas | 2009 | Cochabamba, Bolivia (LMI) | Cross-sectional | FTA-ABS | Determine | Whole Blood | 50 | 1 | 1 | 437 | Pregnant Women |
| 30 | Wang(a) | 2007 | China (LMI) | Cross-sectional | TPHA | VisiTect | Whole Blood | 82 | 30 | 1 | 388 | STD clinic attendees |
| 30 | Wang(b) | 2007 | China (LMI) | Cross-sectional | TPHA | Syphicheck | Whole Blood | 75 | 41 | 1 | 352 | STD clinic attendees |
| 30 | Wang(c) | 2007 | China (LMI) | Cross-sectional | TPHA | SD Bioline | Whole Blood | 102 | 14 | 2 | 351 | STD clinic attendees |
| 30 | Wang(d) | 2007 | China ( LMI) | Cross-sectional | TPHA | Determine | Whole Blood | 83 | 29 | 2 | 387 | STD clinic attendees |
| 30 | Wang(e) | 2007 | China (LMI) | Cross-sectional | TPHA | VisiTect | Whole Blood | 84 | 28 | 0 | 389 | STD clinic attendees |
| 30 | Wang(f) | 2007 | China (LMI) | Cross-sectional | TPHA | Syphicheck | Whole Blood | 82 | 34 | 1 | 352 | STD clinic attendees |
| 30 | Wang(g) | 2007 | China (LMI) | Cross-sectional | TPHA | SD Bioline | Whole Blood | 102 | 14 | 2 | 351 | STD clinic attendees |
| 30 | Wang(h) | 2007 | China (LMI) | Cross-sectional | TPHA | Determine | Whole Blood | 86 | 26 | 0 | 389 | STD clinic attendees |
| 30 | Wang(i) | 2007 | China (LMI) | Cross-sectional | TPHA | VisiTect | Serum | 106 | 6 | 7 | 382 | STD clinic attendees |
| 30 | Wang(j) | 2007 | China (LMI) | Cross-sectional | TPHA | Syphicheck | Serum | 79 | 37 | 4 | 349 | STD clinic attendees |
| 30 | Wang(k) | 2007 | China (LMI) | Cross-sectional | TPHA | SD Bioline | Serum | 111 | 5 | 7 | 346 | STD clinic attendees |
| 30 | Wang(l) | 2007 | China (LMI) | Cross-sectional | TPHA | Determine | Serum | 112 | 0 | 4 | 385 | STD clinic attendees |
| 31 | West | 2002 | Farafenni area of Gambia (LI) | Cross-sectional | TPHA | RST, Quorum Diagnostics,  Vancouver, BC | Serum | 51 | 29 | 41 | 1204 | Women of reproductive age |
| 31 | West | 2002 | Farafenni area of Gambia (LI) | Cross-sectional | RPR + TPHA | RST, Quorum Diagnostics,  Vancouver, BC | Serum | 30 | 10 | 62 | 1223 | Women of  reproductive age |
| 32 | Yang | 2010 | China (LMI) | Case-Control | TPPA titer of 1:80 or above  FTA-Abs | Quantum Dots-Based | Serum | 50 | 0 | 0 | 50 | lab and healthy blood donors |
| 32 | Yang | 2010 | China (LMI) | Case-Control | TPPA titer of 1:80 or above  FTA-Abs | colloidal gold-based  lateral flow test | Serum | 41 | 9 | 0 | 50 | lab and healthy blood donors |
| 33 | Zarakolu | 2002 | Turkey (UMI) | Case-Control | FTA-ABS | ICS | Serum | 13 | 0 | 0 | 124 | STD clinic attendees |

**HI: High Income, UMI: Upper-Middle Income, LMI: Lower-Middle Income, LI: Low Income, TPHA: T. Pallidum haemagglutination assay, TPPA: T. pallidum paticle agglutination assay, FTA-Abs: fluorescent treponemal antibody absorption, RPR: Rapid Plasma Reading test, VDRL: Venereal Disease Research Laboratory test, STD: Sexually Transmitted Diseases, FSW: Female Sex Worker. Determine (Abbott Diagnostics, UK),SD Bioline (Standard, South Korea), Syphicheck (Qualpro, India),and VisiTect (Omega Diagnostics, UK).*

**References:**

1. Benzaken AS, Garcia EG, Sardinha JCG, Dutra Jr JC, Peeling R (2007) Rapid tests for diagnosing syphilis: Validation in an STD clinic in the Amazon Region, Brazil. Cadernos de Saude Publica 23: S456-S464.

2. Benzaken AS, Sabido M, Galban EG, Pedroza V, Vasquez F, et al. (2008) Field evaluation of the performance and testing costs of a rapid point-of-care test for syphilis in a red-light district of Manaus, Brazil. Sexually Transmitted Infections 84: 297-302.

3. Benzaken AS, Sabido M, Galban E, Pedroza V, Araujo AJG, et al. (2011) Field performance of a rapid point-of-care diagnostic test for antenatal syphilis screening in the Amazon region, Brazil. International Journal of STD and AIDS 22: 15-18.

4. Bronzan RN, Mwesigwa-Kayongo DC, Narkunas D, Schmid GP, Neilsen GA, et al. (2007) Onsite rapid antenatal syphilis screening with an immunochromatographic Strip improves case detection and trip treatment in rural south African clinics. Sexually Transmitted Diseases 34: S55-S60.

5. Campos PE, Buffardi AL, Chiappe M, Buendia C, Garcia PJ, et al. (2006) Utility of the Determine Syphilis TP rapid test in commercial sex venues in Peru. Sexually Transmitted Infections 82: v22-v25.

6. Castro AR, Mody HC, Parab SY, Patel MT, Kikkert SE, et al. (2010) An immunofiltration device for the simultaneous detection of non-treponemal and treponemal antibodies in patients with syphilis. Sexually Transmitted Infections 86: 532-536.

7. Castro AR, Esfandiari J, Kumar S, Ashton M, Kikkert SE, et al. (2010) Novel point-of-care test for simultaneous detection of nontreponemal and treponemal antibodies in patients with syphilis. Journal of Clinical Microbiology 48: 4615-4619.

8. Diaz T, Almeida MDB, Georg I, Maia SD, de Souza RV, et al. (2004) Evaluation of the determine rapid syphilis TP assay using sera. Clinical and Diagnostic Laboratory Immunology 11: 98-101.

9. Gianino MM, Dal Conte I, Sciole K, Galzerano M, Castelli L, et al. (2007) Performance and costs of a rapid syphilis test in an urban population at high risk for sexually transmitted infections. 4 ed. Italy. pp. 118-122.

10. Hernandez-Trejo M, Hernandez-Prado B, Uribe-Salas F, Juarez-Figueroa L, Conde-Gonzalez CJ (2006) Maternal and congenital syphilis in two Mexican hospitals: Evaluation of a rapid diagnostic test. Revista de Investigacion Clinica 58: 119-125.

11. Huang QJH, Lan XP, Tong T, Xian W, Chen M, et al. (1996) Dot-immunogold filtration assay as a screening test for syphilis. Journal of Clinical Microbiology 34: 2011-2013.

12. Juarez-Figueroa L, Uribe-Salas F, Garcia-Cisneros S, Olamendi-Portugal M, Conde-Glez CJ (2007) Evaluation of a rapid strip and a particle agglutination tests for syphilis diagnosis. Diagnostic Microbiology and Infectious Disease 59: 123-126.

13. Li J, Zheng HY, Wang LN, Liu YX, Wang XF, et al. (2009) Clinical evaluation of four recombinant Treponema pallidum antigen-based rapid diagnostic tests for syphilis. Journal of the European Academy of Dermatology and Venereology 23: 648-650.

14. Lien TX, Tien NTK, Chanpong GF, Cuc CT, Yen VT, et al. (2000) Evaluation of rapid diagnostic tests for the detection of human immunodeficiency virus types 1 and 2, hepatitis B surface antigen, and syphilis in Ho Chi Minh City, Vietnam. American Journal of Tropical Medicine and Hygiene 62: 301-309.

15. Mabey D, Peeling RW, Ballard R, Benzaken AS, Galban E, et al. (2006) Prospective, multi-centre clinic-based evaluation of four rapid diagnostic tests for syphilis. Sexually Transmitted Infections 82: V13-V16.

16. Miranda AE, Rosetti E, Trindade CR, Gouvea GM, Costa DM, et al. (2009) Prevalence of syphilis and HIV using rapid tests among parturients attended in public maternity hospitals in Vitoria, State of Espirito Santo. Revista Da Sociedade Brasileira de Medicina Tropical 42: 386-391.

17. Mishra S, Naik B, Venugopal B, Kudur P, Washington R, et al. (2010) Syphilis screening among female sex workers in Bangalore, India: comparison of point-of-care testing and traditional serological approaches. Sexually Transmitted Infections 86: 193-198.

18. Montoya PJ, Lukehart SA, Brentlinger PE, Blanco AJ, Floriano F, et al. (2006) Comparison of the diagnostic accuracy of a rapid immunochromatographic test and the rapid plasma reagin test for antenatal syphilis screening in Mozambique. Bulletin of the World Health Organization 84: 97-104.

19. Nessa K, Alam A, Chawdhury FAH, Huq M, Nahar S, et al. (2008) Field evaluation of simple rapid tests in the diagnosis of syphilis. International Journal of STD & AIDS 19: 316-320.

20. Nyamwamu LB, Gicheru MM, Sharma RR, Kimutai A, Tonui WK, et al. (2009) Evaluation of the immunochromatographic strip test for the rapid diagnosis of antenatal syphilis in women in Eldoret, Kenya. Journal of Nanjing Medical University 23: 317-321.

21. Oshiro M, Taira R, Kyan T, Yamane N (1999) Laboratory-based evaluation of DainaScreen TPAb to detect specific antibodies against Treponema pallidum. 1 ed. Japan. pp. 27-32.

22. Rotanov SV, Frigo NV, Kliueva VI (2008) [Comparative study of immunochromatographic sets for rapid diagnosis of syphilis]. Klin Lab Diagn: 42-45.

23. Rotty J, Anderson D, Garcia M, Diaz J, Van de Waarsenburg S, et al. (2010) Preliminary assessment of Treponema pallidum-specific IgM antibody detection and a new rapid point-of-care assay for the diagnosis of syphilis in human immunodeficiency virus-1-infected patients. International Journal of STD & AIDS 21: 758-764.

24. Sano J, Iwakabe A, Ohno H, Aoki Y (1999) Study of anti-Treponema pallidum antibody detection kit <DAINASCREEN-TPAb> employing immunochromatography assay. . 22: 351-354.

25. Sato NS, De Melo CS, Zerbini LCMS, Silveira EPR, Fagundes LJ, et al. (2003) Assessment of the rapid test based on an immunochromatography technique for detecting anti-Treponema pallidum antibodies. 6 ed. Brazil. pp. 319-322.

26. Siedner M, Zapitz V, Ishida M, De la Roca R, Klausner JD (2004) Performance of rapid syphilis tests in venous and fingerstick whole blood specimens. Sexually Transmitted Diseases 31: 557-560.

27. Tinajeros F, Grossman D, Richmond K, Steele M, Garcia SG, et al. (2006) Diagnostic accuracy of a point-of-care syphilis test when used among pregnant women in Bolivia. Sexually Transmitted Infections 82: V17-V21.

28. van Dommelen L, Smismans A, Goossens VJ, Damoiseaux J, Bruggeman CA, et al. (2008) Evaluation of a rapid one-step immunochromatographic test and two immunoenzymatic assays for the detection of anti-Treponema pallidum antibodies. Sexually Transmitted Infections 84: 292-296.

29. Villazon-Vargas N, Conde-Glez CJ, Juarez-Figueroa L, Uribe-Salas F (2009) Evaluation of a rapid diagnostic test to assess the prevalence of maternal syphilis in Bolivia. Revista Medica de Chile 137: 515-521.

30. Wang LN, Yang L, Zheng HY (2007) Clinical evaluation of four recombinant Treponema pallidum antigen-based rapid tests in the diagnosis of syphilis. 4 ed. United Kingdom. pp. 250-253.

31. West B, Walraven G, Morison L, Brouwers J, Bailey R (2002) Performance of the rapid plasma reagin and the rapid syphilis screening tests in the diagnosis of syphilis in field conditions in rural Africa. Sexually Transmitted Infections 78: 282-285.

32. Yang H, Li D, He R, Guo Q, Wang K, et al. (2010) A Novel Quantum Dots-Based Point of Care Test for Syphilis. Nanoscale Research Letters 5: 875-881.

33. Zarakolu P, Buchanan I, Tam M, Smith K, Hook EW (2002) Preliminary evaluation of an immunochromatographic strip test for specific Treponema pallidum antibodies. Journal of Clinical Microbiology 40: 3064-3065.
