# Supplementary figures and images for: Are Treponema pallidum Specific Rapid and Point-of-Care Tests for Syphilis Accurate Enough for Screening in Resource Limited Settings? Evidence from a Meta-Analysis
Source: PLoS One. 2013 Feb 26;8(2):e54695. doi: 10.1371/journal.pone.0054695 (PMC3582640; doi:10.1371/journal.pone.0054695)

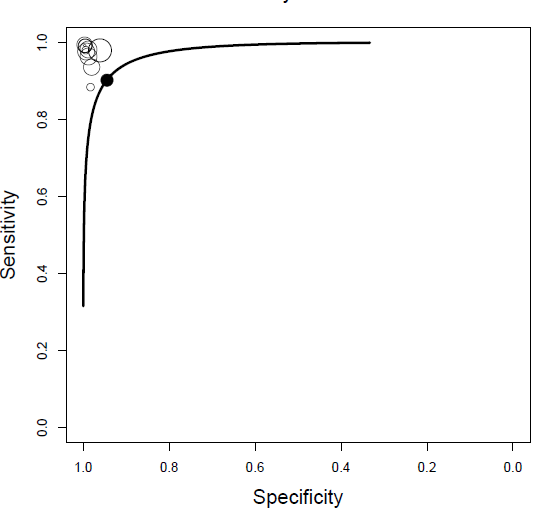

Supplement: Figure S1 — Summary ROC curve for Determine, in serum, using imperfect TP specific reference standard. (TIF) [file pone.0054695.s004.tif]

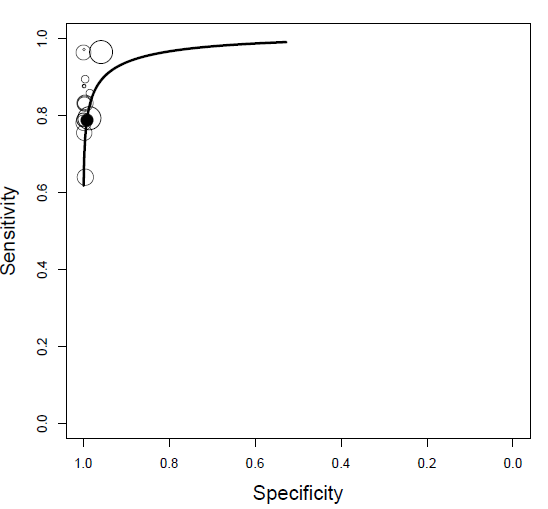

Supplement: Figure S2 — Summary ROC curve for Determine, in whole blood, using imperfect TP specific reference standard. (TIF) [file pone.0054695.s005.tif]

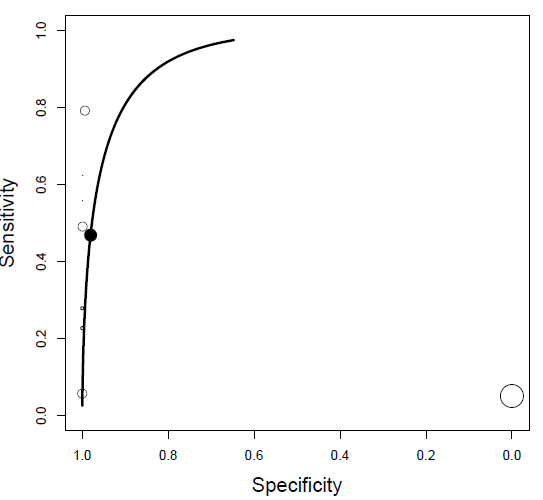

Supplement: Figure S3 — Summary ROC curve for Determine, in whole blood, using imperfect TP and non-TP specific reference standard. (TIF) [file pone.0054695.s006.tif]

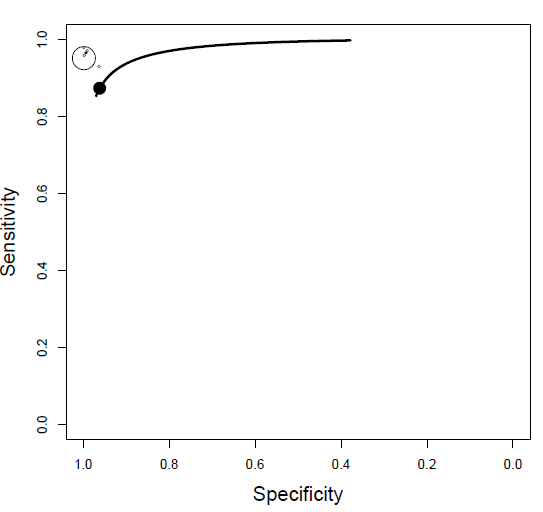

Supplement: Figure S4 — Summary ROC curve for SD Bioline, in serum, using imperfect TP specific reference standard. (TIF) [file pone.0054695.s007.tif]

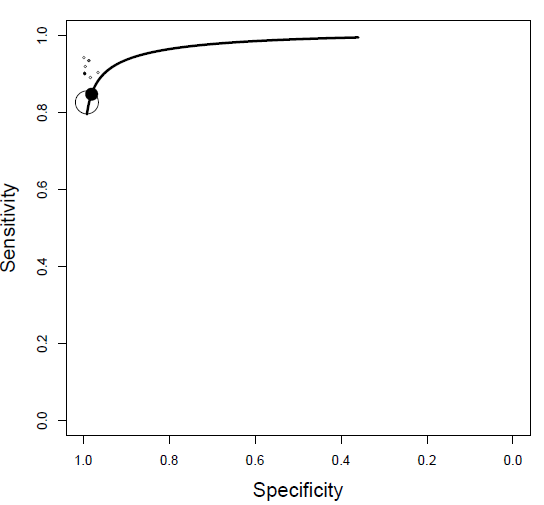

Supplement: Figure S5 — Summary ROC curve for SD Bioline, in whole blood, using imperfect TP specific references standard. (TIF) [file pone.0054695.s008.tif]

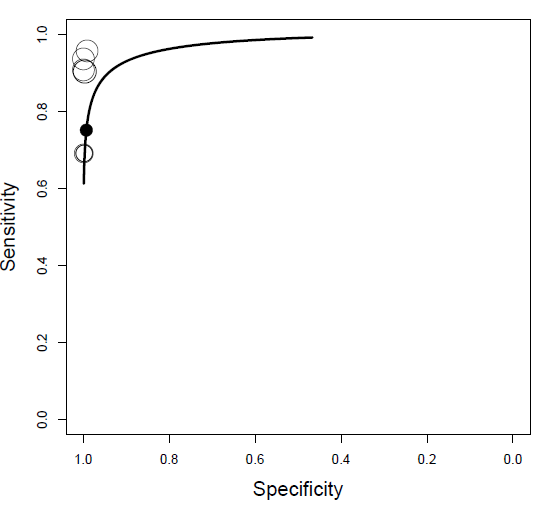

Supplement: Figure S6 — Summary ROC curve for Syphicheck, in serum, using imperfect TP specific reference standard. (TIF) [file pone.0054695.s009.tif]

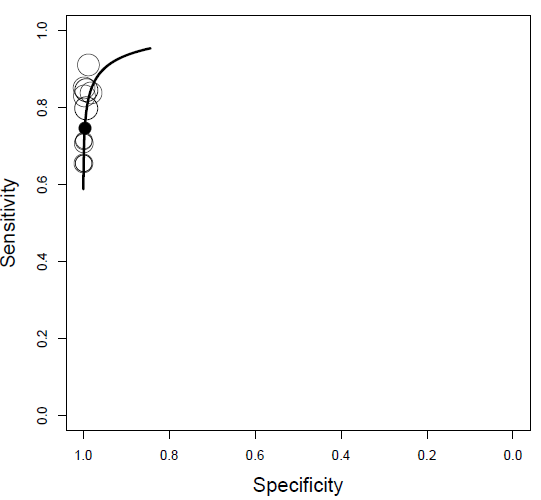

Supplement: Figure S7 — Summary ROC curve for Syphicheck, in whole blood, using imperfect TP specific reference standard. (TIF) [file pone.0054695.s010.tif]

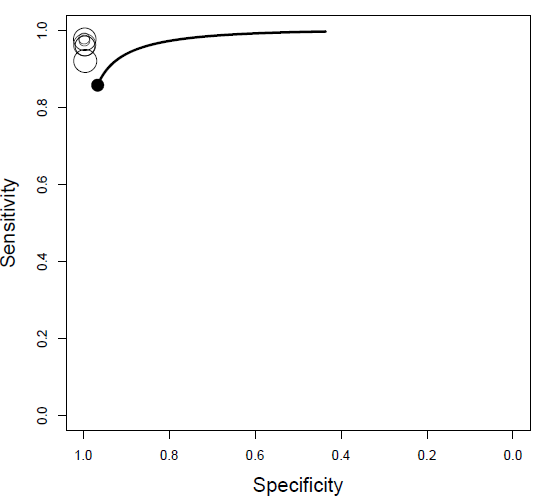

Supplement: Figure S8 — Summary ROC curve for Visitect, in serum, using imperfect TP specific reference standard. (TIF) [file pone.0054695.s011.tif]

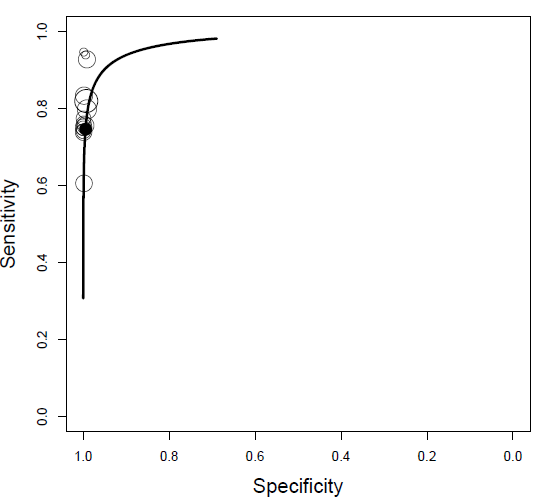

Supplement: Figure S9 — Summary ROC curve for Visitect, in whole blood, using imperfect TP specific reference standard. (TIF) [file pone.0054695.s012.tif]
